# Overusage of Mouse DH Gene Segment, DFL16.1, Is Strain-Dependent and Determined by *cis*-Acting Elements

**DOI:** 10.1155/1994/80207

**Published:** 1994

**Authors:** Michael J. Atkinson, Yenhui Chang, Jakub W. Celler, Carol Huang, Christopher J. Paige, Gillian E. Wu

**Affiliations:** 1 Department of Immunology University of Toronto Ontario Toronto M5S 1A8 Canada; 2 The Wellesley Hospital Research Institute University of Toronto Toronto Canada

**Keywords:** B lineage, DH usage, Ig genes, mouse-strain differences

## Abstract

The DJH structure is of particular importance for diversity in the immunoglobulin heavy chain because it encodes most of CDR3. Here, we investigate mechanisms responsible for generating the DJH structure. We found DFL16.1 was used at a high frequency in normal and transformed pre-B cells (fetal liver > 50%, A-MuLV lines ≅ 25%). One DFL16.1JH1 structure was found repeatedly and was also present in DJH and VDJH databases, suggesting this structure may be conserved in the primary repertoire. Genetic analysis demonstrated that C57BL/6 mice use DFL16.1 in DJH structures more frequently than BALB/c. Examination of individual alleles in (C57BL/6 BALB/c)F1 A-MuLV cell lines revealed that the C57BL/6-derived allele used DFL16.1 twice as often as the BALB/c. This result indicates that part of the mechanism ensuring overusage of DFL16.1 gene segments is *cis*-acting.


*Developmental Immunology*, 1994, Vol 3, pp. 283–295
Reprints available directly from the publisher
Photocopying permitted by license only

© 1994 Harwood Academic Publishers GmbH
Printed in Singapore

### Overusage of Mouse DH Gene Segment, DFL16.1, Is Strain-Dependent and Determined by *cis*-Acting Elements

MICHAEL J. ATKINSON,[‡] YENHUI CHANG, JAKUB W. CELLER, CAROL HUANG, CHRISTOPHER J. PAIGE[‡] and GILLIAN E. WU*[‡]

*Department of Immunology, and ‡The Wellesley Hospital Research Institute, University of Toronto, Toronto M5S 1A8, Canada*

The DJH structure is of particular importance for diversity in the immunoglobulin heavy chain because it encodes most of CDR3. Here, we investigate mechanisms responsible for generating the DJH structure. We found DFL16.1 was used at a high frequency in normal and transformed pre-B cells (fetal liver > 50%, A-MuLV lines ≅ 25%). One DFL16.1JH1 structure was found repeatedly and was also present in DJH and VDJH databases, suggesting this structure may be conserved in the primary repertoire. Genetic analysis demonstrated that C57BL/6 mice use DFL16.1 in DJH structures more frequently than BALB/c. Examination of individual alleles in (C57BL/6×BALB/c)F1 A-MuLV cell lines revealed that the C57BL/6-derived allele used DFL16.1 twice as often as the BALB/c. This result indicates that part of the mechanism ensuring overusage of DFL16.1 gene segments is *cis*-acting.

KEYWORDS: B lineage, DH usage, Ig genes, mouse-strain differences.

### INTRODUCTION

The process of immunoglobulin gene rearrangement is the hallmark of B-cell development. Three sets of gene segments contribute to the variability of immunoglobulin heavy chains, namely, the variable (VH), diversity (DH), and joining (JH) gene segments (Tonegawa, 1983). In the mouse, there are approximately 200–1000 VH segments, 15 DH segments, and 4 JH segments (Kurosawa and Tonegawa, 1982; Brodeur et al., 1984; Winter et al., 1985; Livant et al., 1986; Blankenstein et al., 1987; Lehle et al., 1988; Christoph and Krawinkel, 1989; Ichihara et al., 1989; Kofler et al., 1992). Additional diversity is provided by nucleotide additions (N and P sequences) sometimes associated with the rearrangement process (Desiderio et al., 1984; Feeney, 1990, 1992; Lafaille et al., 1990; Meek 1990; Carlsson et al., 1992). Although there are fewer DH and JH than VH gene segments, the DJH structure contributes significantly to the diversity of the variable portion of immunoglobulin. Together they encode most of the third complementary determining region (CDR3) of the antigen binding site. Understanding the parameters that influence the utilization of particular DJH structures is therefore essential for understanding the generation of immunoglobulin diversity.

Based on sequence similarity, the murine D region can be divided into three families: DQ52 (1 member), Dsp (12 members), and DFL (2 members). DFL16.1 is believed to be the most 5′ D gene segment, whereas DQ52 is the most 3′ gene segment (Kurosawa and Tonegawa, 1982). Several reports have appeared that indicate that the utilization of DH families are different from that expected based on chance alone (Yancopoulos et al., 1988; Nickerson et al., 1989; Feeney, 1990, 1992; Gu et al., 1990; Tsukada et al., 1990; Reynaud et al., 1991; Rolink et al., 1991; Carlsson et al., 1992; Chang et al., 1992). This raises the possibility that selective events, as yet undefined, influence significantly the generation of immunoglobulin diversity (Kohler et al., 1989; Rolink et al., 1991; Kottman et al., 1992). To understand better D gene utilization and to define the genetic parameters that are the basis for biased usage, we have examined DJH structures in primary fetal tissue and cell lines. In this report, we present data demonstrating that the overusage of DFL16.1 in DJH structures is more extensive in

*Corresponding author. Present address; Department of Immunology, University of Toronto, Toronto M5S 1A8, Ontario, Canada

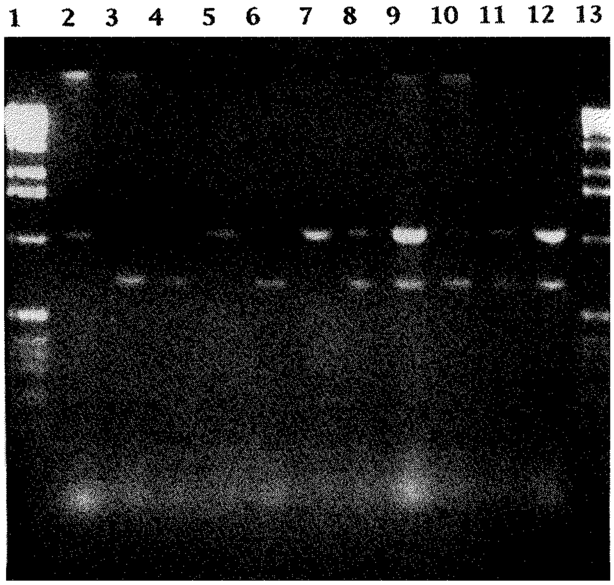

FIGURE 1. Amplification of DFL16.1JH and DSPJH structures: Two hundred fifty ng of DNA from cell lines were amplified singly or in pairs using the standard PCR assay. Lane 1: 1-kb ladder; lane 2: CB135 (DFL16.1JH2); lane 3: CB172(DSPJH3); lane 4: CB134 (DFL16.1JH3); lane 5: CB43 (DSPJH2); lane 6: CB82(DSPJH3); lane 7: CB4(DFL16.1JH2); lane 8: CB43(DSPJH2) and CB82(DSPJH3); lane 9: CB4(DFL16.1JH2) and CB134-(DFL16.1JH3); lane 10: CB172(DSPJH3) and CB135-(DFL16.1JH2); lane 11: CB43(DSPJH2) and CB134(DFL16.1JH3); lane 12: CB4(DFL16.1JH2) and CB82(DSPJH3); and lane 13: 1-kb ladder (BRL).

primary fetal-liver tissue than in lines derived from fetal liver. Data accounting for this observation is consistent with sequential DH to JH rearrangements and with DFL16.1 being a preferred D gene segment in V to DJH joining. Furthermore, we document that one of the determinants responsible for overusage of DFL16.1 is strain-specific and *cis*-acting.

### RESULTS

An understanding of generation of diversity requires the elucidation of the parameters of DH utilization. In BALB/c mice, DFL16.1 is the most 5′ DH gene segment. Thus, DH segment utilization for the $Igh^a$ allele of BALB/c can be estimated by Southern analysis of bands hybridizing specifically to a DH probe using the maps previously determined (Fig. 2) (Tsukada et al., 1990). However, the DH locus in C57BL/6 has not been ordered and thus the relative position of the DH genes in the locus, specifically, DFL16.1, is now known. Therefore, in order to determine DH segment utilization of C57BL/6 $Igh^b$ alleles precisely, a restriction map of the $Igh^b$ D-locus is first needed.

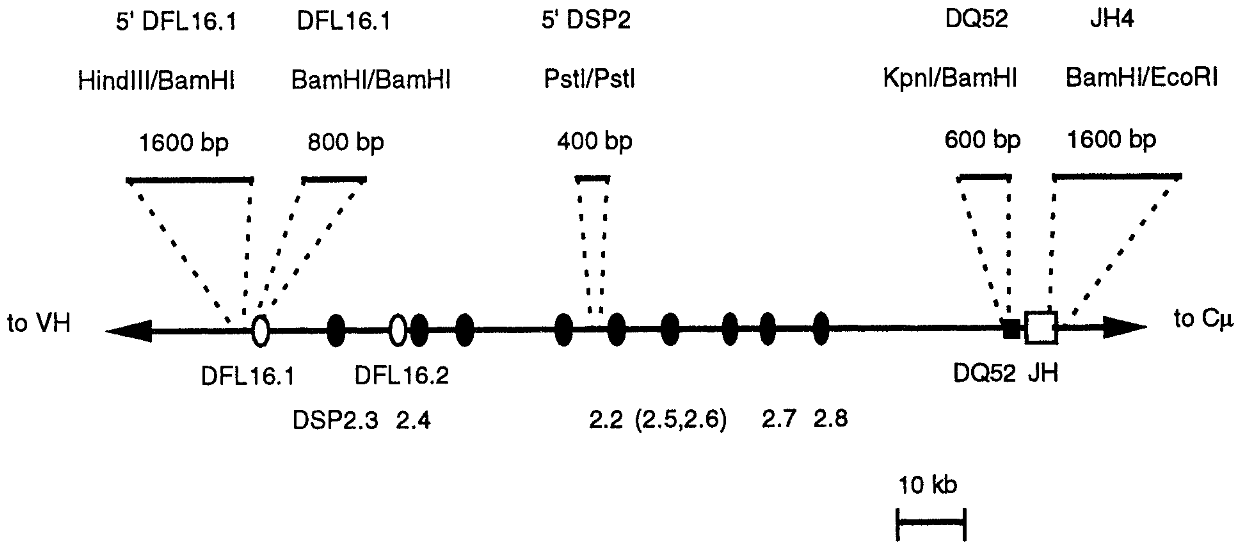

FIGURE 2. D locus in BALB/c: The map of the D region is based on mapping data from Kurosawa and Tonegawa (1982) and Tsukada et al. (1990). The closed box represents DQ52, closed ovals represent DSP family members, and open ovals represent DFL16 family members. The open box indicates the JH region. The probes used in this study, their flanking-restriction endonuclease sites, and their sizes are indicated above the solid bars. The dotted lines indicate the relative position the fragment occupies in the BALB/c genome.

### Mapping the DH Locus on the C57BL/6 $Igh^b$ Allele

Southern analysis of germline DNA from C57BL/6 liver digested with Eco RI and hybridized with DH region probes is demonstrated in Fig. 3. The DFL16.1 probe hybridizes preferentially to a 4.0-kb fragment; the 5′ DFL16.1 and 5′ Dsp probe both hybridize to 4.0-, 5.0-, 5.6-, and 6.0-kb fragments (Fig. 2). In addition, the 5′ DFL16.1 probe detects a 10-kb fragment. Previous studies on the BALB/c allele have shown this fragment does not contain any coding regions for DH gene segments. The DQ52 probe detected a 6.6-kb fragment (which also contains JH locus) (Fig. 3).

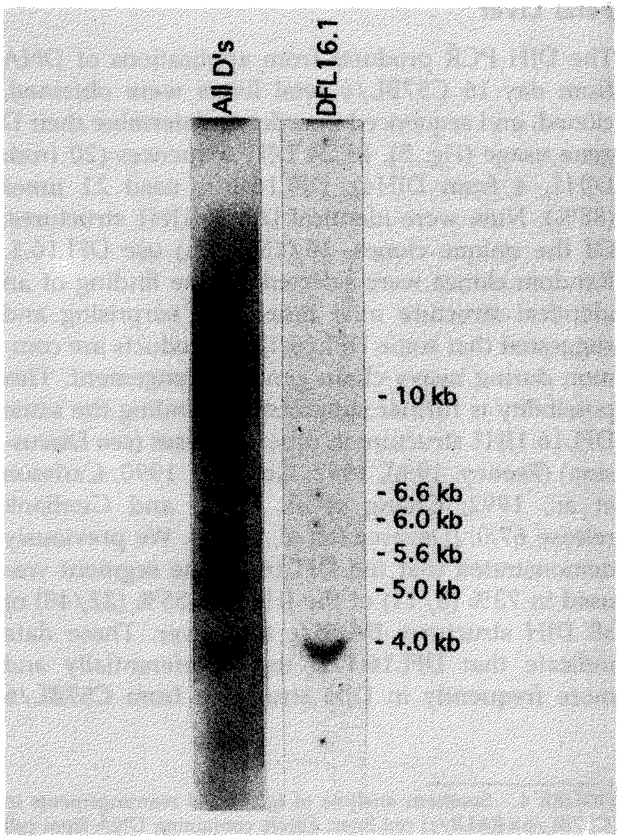

FIGURE 3. Southern analysis of germline DH locus in C57BL/6 DNA: Genomic DNA was extracted from C57BL/6 kidney electrophoresed on 0.8% agarose gel and probed by the method of Southern (1975) (28). The probes used are summarized in Fig. 1. The lane shown on the left was hybridized with a combination of 5′ DFL16.1, 5′ DSP, and DQ52 probes. After exposure, the filter was stripped, exposed, and reprobed with the DFL16.1 probe and is shown in the right-hand lane. Multiple D probes detected germline bands of 4.0-, 5.0-, 5.6-, 6.0-, 6.6-, and 10.0-kb fragments, as indicated. The DFL16.1 probe detected only a 4.0-kb fragment.

Deletion mapping was used to determine the relative map positions of these DH region bands. We selected (C57BL/6×BALB/c)F1 A-MuLV-transformed cell lines that we previously determined to have DJH rearrangements on the $Igh^b$ allele and VDJ rearrangements on the $Igh^a$ allele. Because VDJ rearranged bands are not detected with DH probes under the conditions employed here, the only DH bands detected are the DJH rearrangements on the $Igh^b$ allele. DNA from these cell lines was digested with Eco RI and successively probed with JH- (Fig. 4a) and DH region probes (Figs. 4b and 4c). Unexplainably, the 10-kb fragment is not detectable in some Southern blots and is not here, but previously analysis has confirmed that this band lies 5′ to all DH segments (Atkinson et al., 1991). Some of the hybridizing bands contain multiple DH fragments that are the same size. For example, cell line CB133 (Fig. 3) has all the germline Dsp and DFL hybridizing bands, yet has deleted DQ52 and has a unique DH hybridizing band. The 5.0- and 5.6-kb Dsp/DFL germline bands are more intense than the others and are thus likely to contain multiple DH fragments. A similar state exists in the BALB/c DH locus, where there are multiple 5.0-kb bands containing members of the Dsp family (Ichihara et al., 1989). All D gene segments of size 5.0 kb lie 3′ of the 6.0-kb band as evidenced by the deletion of all 5.0-kb bands in cell lines CB66 and CB73, which have rearranged the 6.0-kb band. It is not possible to determine, in this report, if there are any members of 5.6-kb that lie 3′ of the 6.0-kb band but are too faint to make a noticeable difference in the intensity of the signal of the 5.6-kb bands.

Cell lines CB28 and CB39 have rearranged DH bands (3.6-kb for CB28 and 5.0-kb for CB39) that do not cohybridize with the JH4 probe (Fig. 4). It is not likely that these bands arose as a result of an inversion recombination process as inversions retain intervening DNA and these cell lines have deleted, at least, the 6.6-kb D gene segment. Although it is not known what these structures are, possibilities include D-D fusion (Reynaud et al., 1991) or the introduction of a new Eco RI site. Cell line CB18 (it is a VDJ/VDJ cell line) and cell line CB20 (it has multiple DJs) were excluded from this analysis.

From this analysis (Table 1), the relative map order of C57BL/6 DH gene segments is as follows: 5′ 4.0-kb, 5.6-kb, 6.0-kb, 5.0-kb and 6.6-kb 3′. The 4.0-kb band contains DFL16.1 and, as in the BALB/c genome, DFL16.1 is the most 5′ DH gene segment in the C57BL/6 DH locus.

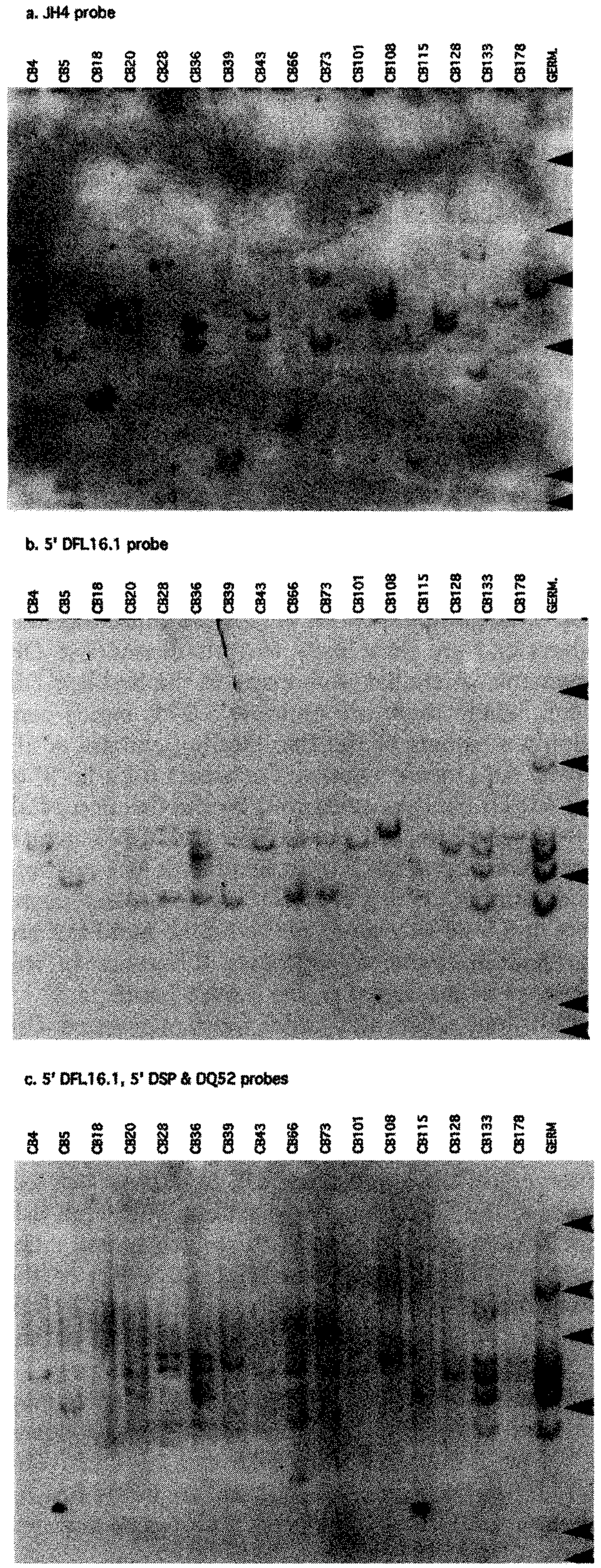

TABLE 1
Summary of D-Region Mapping data for $Igh^b$ Allele

| Cell line | Restriction fragment (kb) 4.0 | 5.6 | 6.0 | 5.0 | 6.6 |
|---|---|---|---|---|---|
| CB 28 | g[a] | g | g | r | d |
| CB 66 | g | g | r | d | d |
| CB 73 | g | g | r | d | d |
| CB 39 | g | r | d | d | d |
| CB 4 | r | d | d | d | d |
| CB 5 | r | d | d | d | d |
| CB 43 | r | d | d | d | d |
| CB 101 | r | d | d | d | d |
| CB 108 | r | d | d | d | d |
| CB 128 | r | d | d | d | d |
| CB 178 | r | d | d | d | d |

[a]D - probe hybridizing bands in germline configuration (g), rearranged (r), or deleted (d).

### DFL16.1 Usage in DJH Structures from Day-16 Fetal Liver

The DJH PCR products from applications of DNA from day-16 C57BL/6 fetal livers were obtained, cloned, and sequenced in order to determine their D gene usage (Fig. 5). In 24 DJH sequences (20 from DJH1, 4 from DJH4), DFL16.1 is used 21 times (88%). Nine were identical DFL16.1JH1 structures. Of the unique clones, 10/13 (77%) use DFL16.1. Random clones were selected, so the finding of an identical structure nine times was surprising and suggested that some DFL16.1JH1 products are common during heavy-chain gene rearrangement. This possibility is further supported by finding the same DFL16.1JH1 structure in other analyses (see Discussion) (Feeney, 1990, 1992; Gu et al., 1990; Carlsson et al., 1992; Chang et al., 1992) and GenBank release 67.0, (Devereux et al., 1984). We previously demonstrated that the DFL16.1 gene segment was used in 73% (8/11) of the JH1 and 55% (22/40) of all DJH structures BALB/c fetal liver. These data indicate that DFL16.1 is used preferentially and more frequently in DJH structures from C57BL/6

FIGURE 4. Southern analysis of $Igh^b$ D-JH rearrangements in (C57BL/6×BALB/c) cell lines: Filters containing DNA from cell lines C57BL/6 kidney DNA (GERM) was hybridized with (a) JH4; (b) 5′ DFL16.1; and (c) a combination of 5′ DFL16.1, 5′ DSP, and DQ52 probes. Cell lines in this study were determined to have a VHDJH rearrangement on the $Igh^a$ allele and a D-JH rearrangement on the $Igh^b$ allele. Cell lines with two major JH hybridizing bands were selected for DH locus-deletion analysis. Relative map order of DH hybridizing bands was determined by comparing bands deleted by rearrangement to germline bands. The filter was stripped and exposed between each probing to ensure complete stripping. The size markers are indicated by arrows at the side of the blots and are shown top to bottom as 23.1, 9.4, 6.6, 4.4, 2.3, and 2.0-kb.

| Clone # | DH | DH sequence | Deleted, D | Deleted, J | JH sequence | JH | Reading frame |
|---|---|---|---|---|---|---|---|
| 47;9;23;11;4;36;3;14;38 | FL16.1 | TT TAT TAC TAC GGT AGT AG | CTAC | | C TAC TGG TAC TTC GAT GTC TGG | 1 | 1 |
| 25 | FL16.1 | TT TAT TAC TAC GGT AGT AGC T | AC | | GC TAC TGG TAC TTC GAT GTC TGG | 1 | 1 |
| 13 | FL16.1 | TT TAT TAC TAC GGT AGT AG | CTAC | CTA | C TGG TAC TTC GAT GTC TGG | 1 | 1 |
| 39 | FL16.1 | TT TAT TAC TAC GGT AGT | AGCTAC | CTAC | TGG TAC TTC GAT GTC TGG | 1 | 1 |
| 17 | FL16.1 | TT TAT TAC TAC GGT AGT AGC T | AC | CTACT | GG TAC TTC GAT GTC TGG | 1 | 1 |
| 30 | FL16.1 | TTT ATT ACT ACG GTA | GTAGCTAC | CTACTGGTACTTC | GAT GTC TGG | 1 | 2 |
| 18;24;48 | FL16.1 | T TTA TTA CTA CGG TAG T | AGCTAC | CTACT | GG TAC TTC GAT GTC TGG | 1 | 3 |
| 10 | FL16.1 | T TTA TTA CTA CGG T | AGTAGCTAC | CTACT | GG TAC TTC GAT GTC TGG | 1 | 3 |
| 41 | SP2.2 | TC TAC TAT GAT TAC GAC | C | TACTGGTAC | TTC GAT GTC TGG | 1 | 1 |
| 45 | SP2.11 | C CTA CTA TAG | GTACGAC | CTAC | TGG TAC TTC GAT GTC TGG | 1 | 3 |
| 15 | FL16.1 | TT TAT TAC TAC GGT AGT AGC T | AC | | AT TAC TAT GCT ATG GAC TAC TGG | 4 | 1 |
| 28;16 | FL16.1 | TT TAT TAC TAC GGT AGT AGC TA | C | ATTACTA | T GCT ATG GAC TAC TGG | 4 | 1 |
| 49 | SP2.3/2.4 | TC TAC TAT GGT TAC | GAC | | AT TAC TAT GCT ATG GAC TAC TGG | 4 | 1 |

FIGURE 5. Sequence of D-JH structures in C57BL/6 fetal liver: DJH structures from C57BL/6 fetal liver from day 16 of gestation were amplified by PCR, cloned, and sequenced, as described in Materials and Methods. 24 D-JH sequences were determined and are summarized here. The clone name, the DH-gene segment, the JH segment, and the reading frame used are indicated. Also shown are the germline sequences that were deleted from the DH and JH borders during the D-JH rearrangement.

fetal liver than BALB/c (21/24 vs. 22/40, $p=0.006$, (Fisher's Exact Test).

Other characteristics of C57BL/6 DJH structures also suggest that selective mechanisms influence their generation. For example, the reading frame (RF) usage is biased to RF1. Eighteen out of 24 (75%) used RF1; only a single clone used RF2 (4%) and 5 used RF3 (21%). Another identifying characteristic is deletions at the DJH boundary (average 7.2 nucleotides) with no "N" additions. These characteristics are similar to those found in BALB/C fetal structures (Gu et al., 1990; Chang et al., 1992) and suggest that a similar element may be responsible for the biased gene usage in both strains (Kottman et al., 1992).

### DFL16.1 Usage in DJH Structures from Fetal-Liver-Derived A-MuLV Lines

Fetal-liver DNA contains the array of DJH structures present at the time the DNA was prepared. The DJH structures could have been in B-cell progenitors at the DJ/DJ stage, VDJ/DJ stage, or perhaps in cells that are not even part of the B lineage. To examine these questions, A-MuLV lines were established and analyzed from (C57BL/6×BALB/c)F1 C57BL/6 and BALB/c fetal-liver cells (day 17 of gestation). All lines were maintained in culture for the same length of time (6 weeks) before DNA was prepared. Lines with DJH and VDJ rearrangements were identified based on Southern blot analysis (described in detail in Atkinson et al., 1991). Forty-eight (C57BL/6×BALB/c)F1, 16 BALB/c, and 14 C57BL/6 VDJ/DJ A-MuLV lines were examined using deletion analysis based on the $Igh^a$ DH locus map (Tsukada et al., 1990) or the $Igh^b$ DH-locus map (Table 2). An example of a DFL16.1JH band is the ≅4.5-kb band in CB5 (Fig. 4). We found that 4/16 (25%) of the DJH rearrangements in the BALB/c lines utilized the DFL16.1 gene segment; 8/14 (57%) in the C57BL/6 lines, and 14/48 (29%) in the (C57BL/6×BALB/c)F1 lines. Because there are 15 identified DH gene segments in the haploid genome, random gene usage would be about 7%. Therefore, DFL16.1 is used in DJH structures significantly more frequently than random usage in the three strains examined and reflect the elevated level of DFL16.1 found in the fetal-liver repertoire.

### DFL16.1 Usage in DJH Structures from $Igh^a$ and $Igh^b$ Alleles

DFL16.1JH structures were more than twice as frequent in C57BL/6 lines as in BALB/c lines (57% vs. 25%). Similarly, fetal-liver DNA from C57BL/6 had more DFL16.1JH structures than BABL/c ($p=0.077$, Fisher's Exact Test). This result suggests that Igh haplotype may influence the rearrangement of DFL16.1. Our set of (C57BL/6×BALB/c)F 1A-MulV cell lines allowed us to determine whether these effects were based on *cis-* or *trans-*acting genetic factors. Of the 48 DJH alleles in the (C57BL/6×BALB/c)F1 cell lines, 30 DJH structures were on the $Igh^a$ allele and 18 on the $Igh^b$ allele. The $Igh^a$ allele used DFL16.1 in 5/30 (17%) of the DJH rearrangements and the $Igh^b$ allele used DFL16.1 in (9/18) 50% of the DJH rearrangements (Table 2). Because there is a significant difference ($p=0.017$, Fisher's Exact Test) between the usage of DFL16.1 in the $Igh^a$ and $Igh^b$ haplotypes, these results suggest that at least some of the factors determining the strain-specific difference in usage act in *cis* on the IgH locus.

### DQ52 Usage in A-MuLV Lines

Previous reports concluded that DQ52 was used frequently due to its proximity to the JH locus (Tsukada et al., 1990). Our analyses of fetal-liver structures of both BALB/c and C57B1/6 mice demonstrated that DQ52JH structures were the second most common DJH structure, accounting about 10%

TABLE 2
DFL16.1 Usage in A-MuLV Lines

| Strain | Ig = gene rearrangemant status | No. of lines | No. of DFL16.1 rearrangements | DFL16.1 usage (%) |
|---|---|---|---|---|
| BALBC | $VDJ^aDJ^a$ | 16 | 4 | 25 |
| C57BL/6 | $VDJ^bDJ^b$ | 14 | 8 | 57 |
| (C57BL/6×BALB/c)F1 | By allele | | | |
| | $VDJ^aDJ^b$ | 18 | 9 | 50 |
| | $VDJ^bDJ^a$ | 30 | 5 | 17 |
| | Summary | | | |
| | VDJ/DJ | 48 | 14 | 29 |

of the DJH structures (Chang et al., 1992). A-MuLV lines allowed us to examine the frequency of DQ52JH structures in situations where much of the rearrangement history of the cell is maintained through "subhaploid" populations (Eisen et al., 1991). Using the DQ52/JH4 PCR primer pair and DNA from 45 (C57BL/6×BALB/c)F1 VDJ/DJ lines and 10 BALB/c VDJ/DJ lines, we found 6 lines (11%) each with a single DQ52JH rearrangement.

VDJ/DJ lines have undergone multiple rearrangements events—D to J, V to DJ, and perhaps secondary D to J rearrangements. Thus, analysis of VDJ/DJ lines may bias the data against DQ52 usage because DQ52JH structures would necessarily be deleted by secondary D to JH replacement. DJ/DJ lines, on the other hand, may be considered to be less mature and may therefore contain an "earlier repertoire" of structures than the VDJ/DJ lines. Using the PCR assay, we analyzed the DH usage of 20 DJ/DJ lines, 13 from BALB/c and 7 from (C57BL/6×BALB/c)F1 fetal livers. All lines had DJH structures amplified by the DSF/JH primer pair but only 1 of the 21 lines had a DQ52JH amplified structure. This line (lane 11 of Fig. 6) had at least seven DJH structures and retained a germline-sized band.

Although, DQ52 has been found to be overused in human fetal cDNAs (Schroeder et al., 1987; Nickerson et al., 1989; Schroeder and Wang, 1990; Mortari et al., 1992) and in an A-MuLV line (Tsukada et al., 1990), our results indicate that overusage of DQ52 may not be a common feature of A-MuLV lines. Thus, proximity to JH alone cannot be the major determinant of DH utilization.

### JH Usage in DFL16.1JH Rearrangements

The overusage of DFL16.1 in DJH structures could be the result of an accumulation of secondary D to

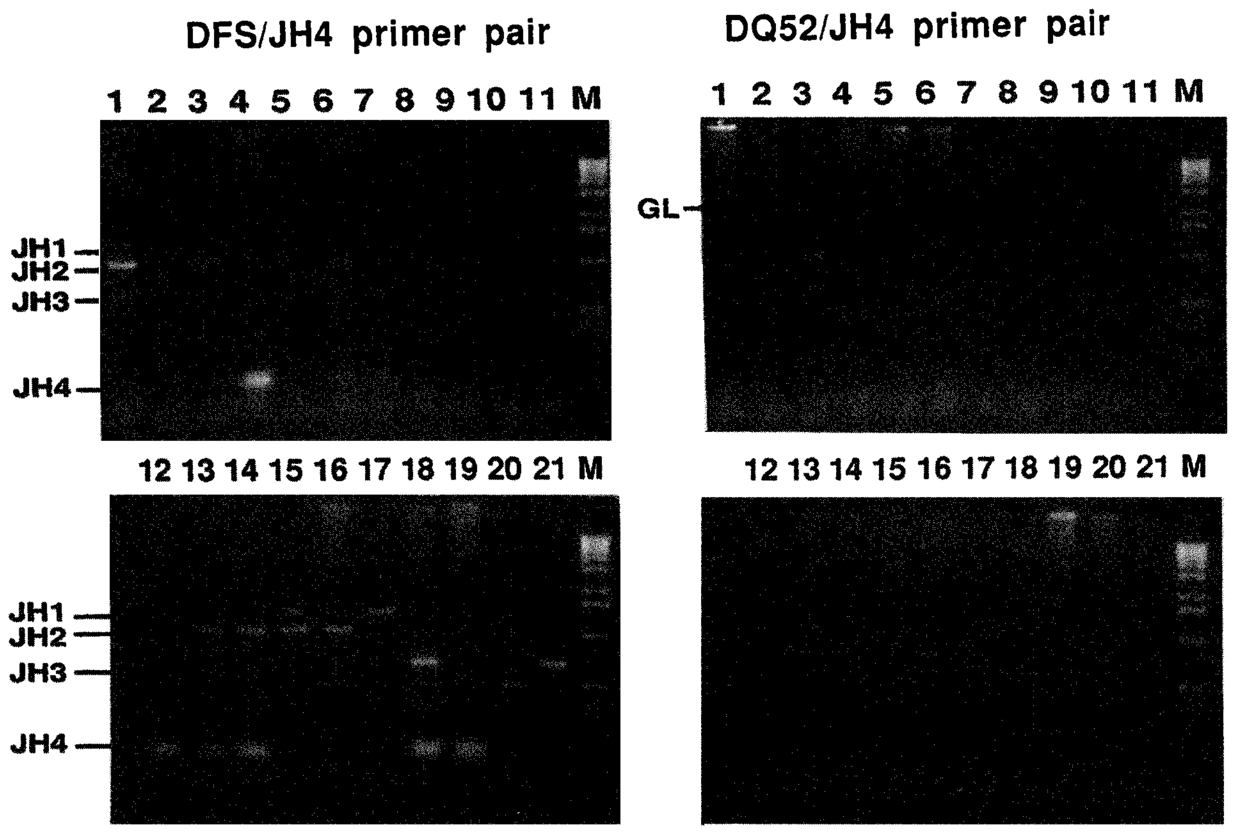

FIGURE 6: Amplification of DNA from A-MuLV lines with DFS/JH4 and DQ52/JH4 primer pairs. 0.5 μg of cell line DNA was amplified as described in the text and the products visualized on an ethidium bromide–stained agarose gel. Markers at the side denote the positions of DJH1 (1.3 kb), DJH2 (1 kb), DJH3 (0.7 kb), and DJH4 (0.13 kb) rearrangements as well as the germline (GL)-sized bands (2 kb). The PCR assay is described in detail in Chang et al. (1992) and in Materials and Methods. Lanes 1–21 are amplifications of 21 A-MuLV lines. Lane M is the 1-kb ladder (BRL).

JH joining events that stop when DFL16.1 is joined to any of the four JHs. Because there are no DH gene segments upstream of DFL16.1, a DFL16.1JH structure cannot undergo DJH replacement events. As a corollary, one would expect to find more DJH3 and DJH4 structures than DJH1 and DJH2 structures in tissues where secondary events were common. We examined our collection of A-MuLV lines for such events by analyzing their JH usage. DNA from each cell line was amplified separately with the DSF/JH and DQ52/JH primer pairs. The amplification products were analyzed by agarose gel electrophoresis. A typical set of reactions is shown in Fig. 6.

Forty-five (C57Bl/6×BALB/c)F1 VDJ/DJ lines were assayed with the DFS/JH4 primer pair. Thirty-six of the lines had visible DJH structures of which 15 had two or more structures. Forty-seven amplified products were detected by ethidium bromide staining. JH1 was used in one of these structures (2%), JH2 in 9 (19%), JH3 in 18 (38%), and JH4 in 19 (40%). There was one unusual structure of 3-kb that was not characterized further. Forty-four (C57BL/6×BALB/c)F1 and 10 (BALB/c) VDJ/DJ lines were assayed with the DQ52/JH4 primer pair. Six lines [5/44 (C57Bl/6×BALB/c)F1 lines, 1/10 BALB/c lines] had one DQ52JH structure each; 1 JH1, 4 JH2, 0 JH3, and 1 JH4. In total for both primer sets, 53 DJH structures were detected. JH1 was used 2/53 (3.8%), JH2 13/53 (25%), JH3 18/53 (34%), and JH4 20/53 (38%) (tabulated in Table 3). The frequency of DJH1 structures is much less than random (25%). This is the result expected if DJH replacement events (which necessarily remove, at least, DJH1 structures) are occurring.

To examine whether these DJH-replacement events result in an accumulation of DFL16.1JH structures and thus are the reason for the overutilization of DFL16.1, we determined the JH usage of DFL16.1JH structures identified by Southern analysis. Of the 12 structures analyzed, 1/12 used JH1, 5/12 JH2, 3/12 JH3, and 3/12 JH4. Of the lines that had a single detectable DJH structure by PCR, 1/1 of the JH1, 5/5 of the JH2, 3/9 of the DJH3, and 3/11 of the DJH4 structures were DFL16.1JH. Because the number of samples is small, we arbitrarily pooled JH1 with JH2 structures and JH3 and JH4 structures. With this small sample size, statistical analysis using Fisher's Exact Test indicates that, given the overall frequency of DFL16.1 usage, the usage of DFL16.1 is not more frequent with JH3 and JH4 than with JH1 and JH2, but rather correlates with JH1 an JH2 usage ($p=0.04$). Thus, although it appears that secondary rearrangement occurs based on the infrequent usage of JH1, it cannot alone account for the level of DFL16.1 because we failed to detect an accumulation of DFL16.1JH3 and DFL16.1JH4 structures. The DFL16.1 gene segment itself may have some inherent characteristics rendering it more recombinogenic and thus more likely to be utilized in D to JH and V to DH rearrangements.

### DISCUSSION

This report shows in two experimental systems and in three mouse strains that a single DH gene segment, DFL16.1, is used in DJH structures at frequencies ranging from 17 to 70%. The overusage of a particular gene segment puts constraints on the degree of diversity in the fetal repertoire, suggesting that there must be underlying advantages to this restrictive overusage. In our effort to determine the reasons for overusage, we ruled out that it could be solely due to secondary D to JH rearrangements and uncovered a strain-specific *cis*-acting genetic element that enhances the overuse of DFL16.1. Moreover, we identified a recurring DJH structure that had counterparts in functional VDJ joins (GenBank version 67), suggesting that this common structure is utilized and that some mechanism has evolved to ensure its perpetuation.

TABLE 3
JH Usage in DJH Structures in VDJ/DJ A-MuLV Lines

| Primer pair | Frequency of lines with amplifiable products | Number of amplifiable products | Amplified products (%) | | | |
|---|---|---|---|---|---|---|
| | | | DJH1 | DJH2 | DJH3 | DJH4 |
| DSF/JH4 | 36/45 | 47 | 1(2.1%) | 9(19%) | 18(38%) | 19(40%) |
| DQ52/JH4 | 6/54 | 6 | 1 | 4 | 0 | 1 |
| Total | | 53 | 2(3.8%) | 13(25%) | 18(34%) | 20(38%) |

### Overusage of DFL16.1 in DJH Structures

Previous analyses of heavy-chain gene rearrangement have generally found overusage of DFL16.1 (Reth et al., 1986; Ichihara et al., 1989; Nickerson et al., 1989; Feeney, 1990; Gu et al. 1990; Tsukada et al., 1990; Rolink et al., 1991; Chang et al., 1992) in normal tissue, transformed and nontransformed lines. For example, Rolink and colleagues have analyzed nontransformed lines derived from fetal-liver cells and found that 50% of the subclones of one line had DFL16JH rearrangements (Rolink et al., 1991). The cumulative data show DFL16.1 usage varying from about 17 to over 70%. The overusage of DQ52 has been found less consistently, notably in the progeny of one A-MuLV line (Tsukada et al., 1990) (where 26% of the usage was DFL16.1 and 25% DQ52), in thymocytes (Suzuki et al., 1989), and in human cDNAs (Nickerson et al., 1989; Schroeder and Wang, 1990; Mortari et al., 1992).

In efforts to understand the mechanisms controlling Ig gene usage, a number of hypothesis has been put forward. These studies have provided many important insights, but have yet to yield a precise mechanism (Holmberg et al., 1986; Kearney and Vakil, 1986; Manser and Gefter, 1986; Wu and Paige, 1988; Kohler et al., 1989; Leclercq et al., 1989; Grandien et al., 1990; Andrade et al., 1991; Tsubata et al., 1991; Kottman et al., 1992; Viale et al., 1992).

Ongoing D to J rearrangements have been detected and studied in A-MuLV lines (for example, Reth et al., 1986). However, as far as we know, there is no documented evidence that they occur in primary tissue. Indeed, analysis of DJH rearrangement in fetal liver and adult bone marrow indicate that many DJs are "blocked" DFL16.1JH1 structures (Gu et al., 1990; Rolink et al., 1991; Chang et al., 1992). Thus, it remains to be seen whether gene replacement is a major contributor to the final DJH usage in primary tissue.

DFL16.1 may be a better substrate for the recombination machinery. The recombination signal sequences themselves may be "better" than those of the other DH gene segments. Although inspection of the RSS of DFL16.1 did not indicate any obvious RSS advantage, subtle differences may be there and these differences remain to be tested. Homologies at joined boundaries—the 3′ end of DFL16.1 and the 5′ end of JH1 are CTAC—have been suggested as a "target" that increases the affinity of DNA/recombinase complex, resulting in preferential joining of these gene segments (Feeney, 1990; Gu et al., 1990; Rolink et al., 1991; Chang et al., 1992). It may be that there are sequences lying 5′ of DFL16.1 that enhance the likelihood of DNA rearrangement in this region of the locus. For example, if a matrix-attachment region (MAR) site lies near DFL16.1, this could render this region more "active" (Dave et al., 1991). Alternatively, there could be sequences 3′ of DFL16.1 repressing recombination and the removal of these sequences through D-JH recombination is required to activate some aspect of B-lineage differentiation. Experiments assaying the DNA flanking DQ52 for protein binding sites have revealed several potential regulatory regions (Kottman et al., 1992). Our observation that there is a further enhancement of DFL16.1 usage due to a *cis*-encoded element indicates that a search for differences in the DH locus among strains, possibly in the vicinity of DFL16.1, might prove fruitful.

### DFL16.1 is Present in VDJH Structures

A genetic mechanism appears to have been developed to ensure DFL16.1 overusage in DJH structures. In order to influence the antibody repertoire, it must be present in VDJ structures. Although it is difficult to compare directly results from A-MuLV lines and results, *ex vivo*, from their tissue of derivation, our analysis of A-MuLV lines and fetal liver suggests a mechanism whereby its presence in VDJs may have been ensured. The DFL16.1 usage in fetal liver was higher (77% in C57BL/6, 55% in BALB/c) than in A-MuLV cell lines (50% in C57BL/6, 17% in BALB/c). Cell lines undergo 50 or more cell divisions during the 6 weeks in culture, whereas the fetal-liver cells probably undergo only 2–6 cell divisions during the period they undergo Ig gene rearrangement (Paige et al., 1984). Given the longer period available for gene rearrangement in culture, preferences for certain DJH structures in V to DJ joining would become evident by comparison of D usage in these two systems. The finding of fewer DFL16.1JH structures in the lines than in fetal liver suggests that more DFL16.1JH structures have been used in V to DJ joining and thus a DFL16.1JH structure may be a preferred substrate for VH to DJ joining.

As well as the data presented in this report, this hypothesis is supported by data showing that DFL16.1 is overused in VDJH structures from fetal or newborn tissue. Feeney found DFL16.1 was used in 33 out of 114 (30%) VDJH cDNA structures from newborn BALB/c spleen (Feeney, 1990). Gu and coworkers found DFL16.1 was used in 32 out of 72 (44%) of VDJH segment in C.B-20 pre-B and B cells (Gu et al., 1991b). If this overusage has relevance for B-cell, differentiation and the generation of diversity, one might expect to find common structures in unrelated individuals. At birth, the Ig repertoire includes specificities that will protect against common pathogens. In addition, the Ig repertoire may be a result of the development of a connectivity of idiotype/antiidiotype specificities (Kearney and Vakil, 1986; Carlsson and Holmberg, 1990; Hirashima et al., 1990; Carlsson et al., 1991). In both cases, certain recombinations of VH, D, and JH gene segments may be necessary to generate the desired specificities or "connecting" idiotypes. Evidence that this indeed may be the case comes from the identification of the same DFL16.1JH1 structure in 9 out of 20 C57Bl/6 DJH1 structures, in 7 out of 11 BALB/c DJH1 structures (Chang et al., 1992), in 3 out of 8 of the newborn VDJH1 sequences of Gu and coworkers (Gu et al., 1990), and in 5 out of 20 VDJH1 structures from newborn tissue (yet only 4 out of 55 VDJH1 structures from adult tissues) of Feeney (1990). Moreover, a retrovirus budding B lymphoma has the same DJH join in its antigen receptor (Jack et al., 1992). A search of GenBank (release 67) identified 12 such rearranged VDJH1 gene structures. Interestingly, some of these structures had antiphosphorylcholine specificity, an antigen specificity present in the newborn repertoire. The frequency of this joint and its presence in the functional Ig repertoire reinforces the notion that this structure is important and thus its presence has been ensured by selection during the evolution of the species.

### Strain Differences

In the study reported here, there is significantly more DFL16.1 gene segment usage in DJH structures from $Igh^b$ alleles than $Igh^a$ alleles. One possible explanation for this may be that DSP2 structures are preferred substrates for VDJH structures on $Igh^b$ alleles, and therefore are underrepresented in DJH structures. A prediction of this model would be an overrepresentation of DSP2 segments in VDJH structures on $Igh^b$ alleles. To date, there has been documentation of DFL16.1 and not DSP2 overusage on $Igh^b$ alleles (Gu et al., 1991b).

Strain differences in VH gene rearrangements have been documented (Cancro and Klinman, 1981; Wu and Paige, 1986; Jeong et al., 1988; Yancopoulos et al., 1988; Freitas et al., 1989; Atkinson et al., 1991; Kofler et al., 1992; Viale et al., 1992). In general, C57BL/6 mice have been found rearranged to 5′ VH-gene segments more frequently than BALB/c mice. Thus, for both DH- and VH-gene segments, C57BL/6 mice rearrange Ig genes more frequently to the 5′ region of the locus. Because it is not known whether the distance between DFL16.1 and JH is the same in both strains, it remains possible that there is a difference in this distance. If there is a difference, this difference either because of the DNA sequence therein or because of the distance per se may be part of the reason for the strain difference in DFL16.1 usage.

Strain differences in DH-gene usage are also seen in antibody responses against defined antigens. One well-described system is the antibody response against the hapten NP. C57BL/6 mice immunized with NP produce antibodies with the idiotype $NP^b$; BALB/c mice are not able to produce this idiotype. The congenic mouse C-B/R3 that has the $Igh^b$ VH and $\lambda$ locus and the $Igh^a$ D-J locus has a reduced frequency of the $NP^b$ idiotype (Klinman and Linto, 1988). This idiotype contains DFL16.1 and because the coding nucleotides for DFL16.1 are identified in BALB/c and C57BL/6, this result indicates that a genetic element associated with the $Igh^a$ D-J region must be influencing the frequency of cells bearing this specific CDR3. This *cis*-acting element may be the same or similar to the element described in this study.

### MATERIALS AND METHODS

### Mice and Cell Lines

Mice were purchased from Jackson Laboratories (Bar Harbor, ME) and maintained at the animal colony of the Ontario Cancer Institute. Timed pregnancies were established as previously described, with day 0 of gestation being the day of mating. Livers were removed from fetuses at day 12 to 17 of gestation (Paige et al., 1984). Six to eight fetal livers from one mother were pooled. Lines were derived from 17-day fetal-liver cells of BALB/c, C57BL/6, and (C57BL/6×BALB/c)F1 fetuses infected with Abelson murine leukemia virus (A-MuLV), as described previously (Atkinson et al., 1991). Cell lines were cultured for a maximum of 6 weeks *in vitro* prior to analysis of gene rearrangements.

### DNA Preparation

Single-cell suspensions were prepared from fetal liver using standard procedures (Paige et al., 1984). Genomic DNA was isolated from fetal-liver cells or cultured cells as described previously (Atkinson et al., 1991) with the following modification: Precipitated DNA was spooled onto glass rods, washed in 70% ethanol, and resuspended in TE buffer (Maniatis et al., 1982).

### Southern Analysis of Ig-Gene Rearrangements

The method used to determine of the immunoglobulin rearrangement status of individual alleles in A-MuLV cell lines is described in detail elsewhere (Atkinson et al., 1991). Briefly, DNA from cell lines is electrophoresed through 0.8% agarose TAE gels (Maniatis et al., 1982), transferred to Zeta-Probe membranes (Bio-Rad), and analyzed using the method of Southern with DH and JH4 segment probes as indicated (Southern, 1975). Four DH probes were used. The DFL16.1 probe is an 800-bp *BamHI-BamHI* fragment that contains DFL16.1 coding and flanking sequences. The 5′ DFL16.1 probe is a 1600-bp *HindIII-BamHI* fragment located 5′ to DFL16.1 coding region. The 5′ DSP2 fragment is a 400-bp *PstI-PstI* fragment located 5′ to DSP2.2. The DQ52 probe is a 600-bp *KpnI-BamHI* fragment containing DQ52 coding and flanking sequences. The JH probe is a 1200-bp *HindIII-EcoRI* containing JH4 coding and flanking sequences. The blots were hybridized and washed as described (final wash was 0.1%SDS, 1×SSC, 65°C, 30 min) and exposed to X-ray film with intensifying screens for 1–7 days (Atkinson et al., 1991).

### Oligonucleotide Primers

The DSF primer is 5′ AGGGATCCTTGTGAAGG-GATCTACTACTGTG 3′. It is a 31 mer extending from the 5′ end of the recombination signal sequence (RSS) nonamer of DSP/DFL through the spacer to the 3′ end of the heptamer, and contains no DH-coding sequences. It is specific for DFL- and Dsp-gene segments, differing from the published sequences of each member of the two families in three positions. The DQ52 primer is 5′ GCGGAG-CACCACAGTGCAACTGGGAC 3′. It is a 26 mer, specific for DQ52, extending from within the 5′ RSS spacer through the heptamer to the end of the coding sequence of DQ52. It is a unique sequence in GenBank. The JH4 primer is 5′ AAAGACCTGCA-GAGGCCATTCTTACC 3′. It is a 26 mer containing sequences in the J-C intron immediately 3′ of JH4 exon excepting that the ninth nucleotide was changed from a C to a G to obtain a *PstI* site. It is a unique sequence in GenBank.

The oligonucleotides were synthesized on a DNA synthesizer (Applied Biosystems) and purified by using NENSORB PREP cartridges (Du Pont).

### PCR Assay

The assay for DJH1 to DJH4 rearrangements was developed and standardized as reported in (Chang et al., 1992). In summary, PCR reactions were performed in siliconized 500-$\mu$l epindorf tubes in a volume of 100 $\mu$l in 10 mM Tris-Cl (pH 8.3 at 25°C), 50 mM KCl, 1.5 mM $MgCl_2$, 0.01% (w/v) gelatin, and containing 0.5 $\mu$g DNA, 200 $\mu$M of each dNTP, 0.5 $\mu$M of each oligonucleotide primer, and 2.5 units Taq polymerase (Perkin- Elmer Cetus). The reaction was overlaid with oil. A Perkin-Elmer Cetus DNA thermo-cycler was used. Each cycle consisted of 1-min denaturation at 94°C, 1.5-min annealing at 60°C, and 2-min polymerization at 72°C. The cycle was repeated 30 times. The polymerization time was extended an additional 3 sec in each cycle. The final polymerization step was extended an additional 10 min. One-tenth of each PCR amplification reaction was loaded on a 1.5% agarose gel (Sigma), electrophoresed in TAE buffer (Maniatis et al., 1982), stained with ethidium bromide, and visualized for photography. Experiments designed to ensure that amplification was both quantitative and qualitative for the DSF/JH4 and the DQ52/JH4 primer pairs are described in detail in Chang et al., 1992. For example, the DSF primer anneals to both Dsp and DFL16.1 gene segments; therefore, it was necessary to determine whether amplification was independent of the DH gene to which the primer had annealed. This was confirmed as follows (Fig. 1): VDJ/DJ lines using either a Dsp or DFL16.1 gene segment on the DJH allele were identified (as described in what follows). DNA from lines having DJH2 and DJH3 rearrangements were amplified singly or in pairs at 1:1 ratios in the amounts of 250 ng each per reaction. As shown in Fig. 1, the degree of amplification of each DJH rearrangement either singly or paired was independent of whether the DNA contained DFL16.1JH or DspJH sequences, verifying that the DSF primer does not amplify DFL16.1 preferentially. The differences in the intensity of coamplified bands from the cell lines may be due to differences in DNA preparations, leading us to now preexamine DNA before amplification. The standardization for the DQ52/JH4 pair is described in Materials and Methods and Fig. 5 of Chang et al., 1992.

### Cloning and Sequencing

The amplified products were purified from Nusieve agarose (FMC Bioproducts) gels and cloned into pBlueScribe either by blunt ligation or by utilizing the *BamHI* and *PstI* enzyme sites contained in the primers. Sequencing was performed using the double-stranded method with the T7 Sequencing kit (Pharmacia) and the reverse universal sequencing primers.

### Statistics

Statistical comparisons in 2×2 contingency tables were made using Fisher's Exact Test.

### ACKNOWLEDGMENTS

We thank Calvin Yu, Dale Ramsden, and Jacqueline Pennycook for helpful discussion. We thank Caren L. Furlonger and R. Ian McDermott for excellent technical assistance and Lynne Omoto for careful preparation of the manuscript. This work was supported by grants to CJP and GEW from the Medical Research Council of Canada and the National Cancer Institute of Canada Terry Fox Marathon of Hope. GEW is an MRC scholar; MJA, a recipient of an Ontario Graduate Scholarship and an Arthritis Society of Canada Fellowship.

*(Received May 24, 1993)*

*(Accepted July 16, 1993)*

### REFERENCES

Andrade L., Huetz F., Poncet P., Thomas-Vaslin V., Goodhardt M., and Coutinho A. (1991). Biased VH gene expression in murine CD5 B cells results from age-dependent cellular selection. Eur. J. Immunol. **21**: 2017–2023.

Atkinson M.J., Michnick D.A., Paige C.J., and Wu G.E. (1991). Immunoglobulin gene rearrangements on individual alleles of A-MuLV cell lines from (C57BL/6×BALB/c)F1 fetal livers. J. Immunol. **146**: 2805–2812.

Blankenstein T., Bonhomme F., and Krawinkel U. (1987). Evolution of pseudogenes in the immunoglobulin VH-gene family of the mouse. Immunogenetics **26**: 237–248.

Brodeur P., Thompson M., and Riblet R. (1984). The content and organization of the mouse Igh-V families. *In*: Regulation of the immune system, Cantor H. Chess L., and Sercarz E., Eds. (New York: Alan R. Liss), pp. 445–453.

Cancro M.P., and Klinman N.R. (1981). B cell repertoire ontogeny: Heritable but dissimilar development of parental and F1 repertoires. J. Immunol. **126**: 1160–1164.

Carlsson L., Andersson A., and Holmberg D. (1991). Germ-line origin of functional idiotype interactions: Identification of two idiotypically connected, natural antibodies that are encoded by germ-line elements. Eur. J. Immunol. **21**: 2285–2288.

Carlsson L., and Holmberg D. (1990). Genetic basis of the neonatal antibody repertoire: Germline V-gene expression and limited N-region diversity. Int. Immunol. **2**: 639–643.

Carlsson L., Övermo C., and Holmberg D. (1992). Selection against N- region diversity in immunoglobulin heavy chain variable regions during the development of pre-immune B cell repertoires. Int. Immunol. **4**: 540–553.

Chang Y., Paige C.J., and Wu G.E. (1992). Enumeration and characterization of DJH structures in mouse fetal liver. EMBO J. **11**: 1891–1899.

Christoph T., and Krawinkel U. (1989). Physical linkage of variable, diversity and joining gene segments in the immunoglobulin heavy chain locus of the mouse. Eur. J. Immunol. **19**: 1521–1523.

Dave V.P., Modak M.J., and Pandey V.N. (1991). Nuclear matrix bound V(D)J recombination activity in rat thymus nuclei: An in vitro system. Biochemistry **30**: 4763–4767.

Desiderio S.V., Yancopoulos G.D., Paskind M., Thomas E., Boss M.A., Landau N., Alt F.W., and Baltimore D. (1984). Insertion of N regions into heavy-chain genes is correlated with expression of terminal deoxynucleotidyl transferase in B cells. Nature **311**: 752–755.

Devereux, Haeberli, and Smithies. (1984). A comprehensive set of sequence analysis programs for the VAX. Nucleic Acids Res. **12**: 387–395.

Eisen A., Atkinson M.J., Celler J.C., and Wu G.E. (1991). DJ/DJ A-MuLV pre-B cell lines have more recombinase activity than VDJ/VDJ lines. Int. Immunol. **3**: 477–484.

Feeney A.J. (1990). Lack of N regions in fetal and neonatal mouse immunoglobulin V-D-J junctional sequences. J. Exp. Med **172**: 1377–1390.

Feeney A.J. (1992). Predominance of VH-D-JH junctions occurring at sites of short sequence homology results in limited junctional diversity in neonatal antibodies J. Immunol. **149**: 222–229.

Freitas A.A., Lambezat M.P., and Coutinho A. (1989). Expression of antibody V-regions is genetically and developmentally controlled and modulated by the B lymphocyte environment. Int. Immunol. **1**: 342–354.

Grandien A., Coutinho A., and Andersson J. (1990). Selective peripheral expansion and activation of B-cells expressing endogenous immunoglobulin in μ-transgenic mice. Eur. J. Immunol. **20**: 991–998.

Gu H., Förster I., and Rajewsky K. (1990). Sequence homologies, N sequence insertion and JH gene utilization in VHDJH joining: Implications for the joining mechanism and the ontogenic timing of Lyl B cell and B-CLL progenitor generation. EMBO J. **9**: 2133–2140.

Gu H., Kitamura D., and Rajewsky K. (1991a). B cell development regulated by gene rearrangement: Arrest of maturation by membrane-bound Dμ protein and selection of DH element reading frames. Cell **65**: 47–54.

Gu H., Tarlinton D., Muller W., Rajewsky K., and Förster I. (1991b). Most peripheral B cells in mice are ligand selected. J. Exp. Med. **173**: 1357–1371.

Hirashima K., et al. (1990). High idiotypic connectivity of the VH7183-encoded antibodies directed to a murine embryonic carbohydrate antigen, Lewis-Y, as ascertained by syngeneic anti-idiotypic monoclonal antibodies. J. Immunol. **145**: 224–232.

Holmberg D., Freitas A.A., Portnoi D., Jacquemart F., Avrameas S., and Coutinho A. (1986). Antibody repertoires of normal BALB/c mice: B lymphocyte populations defined by state of activation. Immunol. Rev. **93**: 147–169.

Ichihara Y., Hayashida H., Miyazawa S., and Kurosawa Y. (1989). Only DFL16, DSP2 and DQ52 gene families exist in mouse immunoglobulin heavy chain diversity gene loci, of which DFL16 and DSP2 originate from the same primordial DH gene. Eur. J. Immunol. **19**: 1849–1854.

Jack H.M., Beck-Engleser G., Lee G., Wofsy D., and Wabl M. (1992). Tumorigenesis mediated by an antigen receptor. Proc. Nat. Acad. Sci. USA **89**: 8482–8486.

Jeong H.D., Komisar J.L., Kraig E., and Teale J.M. (1988). Strain dependent expression of VH gene families. J. Immunol. **140**: 2436–2441.

Kearney J.F. and Vakil M. (1986). Idiotype directed interactions during ontogeny play a major role in the establishment of the adult B-cell repertoire. Immunol. Rev. **94**: 39–50.

Klinman N.R., and Linto P.J. (1988). The clonotype repertoire of B-cell subpopulations. Adv. Immunol. **42**: 1–93.

Kofler R., Stephan G., Kofler H., and Helmberg A. (1992). Mouse variable-region gene families: Complexity, polymorbism and use in non-autoimmune responses. Immunol. Rev. **128**: 5–21.

Kohler G., Iglesias A., Lamers R., Kopf M., Buhler B., and Fritzche U. (1989). Regulation of immunoglobulin gene rearrangement. *In*: Progress in immunology, Melchers. F., Ed. (Berlin: Springer), vol. VII, pp. 324–330.

Kottman A.H., Brack C., Eibel H., and Kohler G. (1992). A survey of protein- DNA interaction sites within the murine immunoglobulin heavy chain locus reveals a particularly complex pattern around the DQ52 element. Eur. J. Immunol. **22**: 2113–2120.

Kurosawa Y., and Tonegawa S. (1982). Organization, structure and assembly of immunoglobulin heavy chain diversity DNA segments. J. Exp. Med. **155**: 201–218.

Lafaille J.J., DeCloux A., Bonneville M., Takagaki Y., and Tonegawa S. (1990). Junctional sequences of T cell receptor g/d genes: Implication for gd T cell lineages and for a novel intermediate of V-(D)-J joining. Cell **59**: 859–870.

Leclercq L., Butkeraitis P., and Reth M. (1989). A novel germ-line $J_\kappa$ transcript starting immediately upstream of $J_\kappa 1$. Nucleic Acids Res. **17**: 6809–6819.

Lehle G., Kolb C., Kappen C., Schuppel R., Weiler E., and Krawinkel U. (1988). A map of VH genes located next to the DH region in the Igh locus of two congenic Igh-recombinant inbred mouse strains. Eur. J. Immunol. **18**: 1275–1281.

Livant D., Blatt C., and Hood L. (1986). One heavy chain variable region gene segment subfamily in the BALB/c mouse contains 500–1000 or more members. Cell **47**: 461–470.

Maniatis T., Fritsch E.F., and Sambrook J. (1982). Molecular cloning: A Laboratory Manual. *In*: Eds. (Cold Spring Harbor, NY: Cold Spring Habor Laboratory), pp. 000–000.

Manser T., and Gefter M.L. (1986). The molecular evolution of the immune response: Idiotope-specific suppression indicates that B cells express germ- line-encoded V genes prior to antigenic stimulation. Eur. J. Immunol. **16**: 1439–1444.

Meek K. (1990). Analysis of junctional diversity during B lymphocyte development. Science **250**: 820–823.

Mortari F., Newton J.A., Wang J.Y., and Schroeder Jr. H.W. (1992). The human cord blood antibody repertoire. Frequent usage of the VH7 gene family. Eur. J. Immunol. **22**: 241–245.

Nickerson K., Berman J., Glickman E., Chess L., and Alt F. (1989). Early human IgH gene assembly in Epstein-Barr virus transformed fetal B cell lines. J. Exp. Med. **169**: 1391–1403.

Paige C.J., Gisler R.H., McKearn J.P., and Iscove N.N. (1984). Differentiation of murine B-cell precursors in agar culture. Frequency, surface marker analysis and requirements for growth of clonable pre-B cells. Eur. J. Immunol. **14**: 979–987.

Reth M., Jackson S., and Alt F. (1986). VHDJH formation and DJH replacement during pre-B differentiation: Nonrandom usage of gene segments. EMBO J. **5**: 2131–2138.

Reynaud C.A., Anquez V., and Weill J.-C. (1991). The chicken D locus and its contribution to the immunoglobulin heavy chain repertoire. Eur. J. Immunol. **21**: 2661–2670.

Rolink A., Kudo A., Karasuyama H., Kikuchi Y., and Melchers F. (1991). Long term proliferating early pre B cell lines and clones with the potential to develop to surface Ig-positive, mitogen reactive B cells *in vitro* and *in vivo*. EMBO J. **10**: 327–336.

Schroeder Jr. H., Hillson J., and Perlmutter R. (1987). Early restriction of the human antibody repertoire. Science **238**: 791–793.

Schroeder Jr. H., and Wang J.Y. (1990). Preferential utilization of conserved immunoglobulin heavy chain variable gene segments during human fetal life. Proc. Natl. Acad. Sci. USA **87**: 6146–6150.

Schroeder Jr. H.W., Walter M.A., Hofker M.H., Ebens A., Van Dijik K.W., Cox D.W., Milner E.C.B., and Perlmutter R.M. (1988). Physical linkage of an immunoglobulin heavy chain gene segment to diversity and joining segments. Proc. Natl. Acad. Sci. USA **85**: 8196–8200.

Schuler W., Ruetsch N.R., Amsler M., and Bosma M.J. (1991). Coding joint formation of endogeneous T cell receptor genes in lymphoid cells from SCID mice: Unusual P-nucleotide additions in VJ-coding joints. Eur. J. Immunol. **21**: 589–595.

Southern E.M. (1975). Detection of specific sequences among DNA fragments separated by gel electrophoresis. J. Mol. Biol. **98**: 503–507.

Suzuki H., Abe M., Shin-ichi N., Nakayama E., and Shiku H. (1989). Preferential usage of JH2 in D-J joinings with DQ52 in murine lymphocytes. Int. Immunol. **1**: 643–646.

Tonegawa S. (1983). Somatic generation of antibody diversity. Nature **302**: 575–581.

Tsubata T., Tsubata R., and Reth M. (1991). Cell surface expression of the short immunoglobulin m chain (Dμ protein) in murine pre-B cells is differently regulated from that of the intact μ chain. Eur. J. Immunol. **21**: 1359–1363.

Tsukada S., Sugiyama H., Oka Y., and Kishimoto S. (1990). Estimation of D- segment usage in initial D to JH joinings in a murine immature B cell line. Preferential utilization of DFL16.1, the most 5′ D segment and DQ52, the most JH-proximal D segment. J. Immunol. **144**: 4053–4059.

Viale A.-C., Coutinho A., and Freitas A.A. (1992). Differential expression of VH gene families in peripheral B-cell repertoires of newborn or adult IgH congenic mice. J. Exp. Med. **175**: 1449–1465.

Winter E., Radbruch A., and Krawinkel U. (1985). Members of novel VH gene families are found in VDJ regions of polyclonally activated B-lymphocytes. EMBO J. **11**: 2861–2867.

Wu G.E., and Paige C.J. (1986). VH gene family utilization in colonies derived from B and pre-B cells detected by the RNA colony blot assay. EMBO J. **5**: 3475–3481.

Wu G.E., and Paige C.J. (1988). VH gene family utilization is regulated by a locus outside of the VH region. J. Exp. Med. **167**: 1499–1504.

Yancopoulos G.D., Malynn B.A., and Alt F.W. (1988). Developmentally regulated and strain specific expression of VH gene families. J. Exp. Med. **168**: 417–435.

